# Telemedicine Adoption and Prospects in Sub-Sahara Africa: A Systematic Review with a Focus on South Africa, Kenya, and Nigeria

**DOI:** 10.3390/healthcare13070762

**Published:** 2025-03-29

**Authors:** Abayomi O. Agbeyangi, Jose M. Lukose

**Affiliations:** Department of Business and Application Development, Walter Sisulu University, East London 5200, South Africa; jlukose@wsu.ac.za

**Keywords:** telemedicine, digital health solutions, healthcare technology, Sub-Saharan Africa

## Abstract

**Background/Objectives:** Telemedicine has emerged as a transformative solution to healthcare access challenges in Sub-Saharan Africa, where many populations remain underserved. This systematic review focuses on the adoption, implementation, and technological prospects of telemedicine in South Africa, Kenya, and Nigeria, three countries leading the region in healthcare innovations. **Methods:** A systematic search of PubMed, Scopus, and Web of Science, guided by PRISMA protocols, identified 567 studies published between 2014 and 2024, of which 53 met the inclusion criteria with a focus on telemedicine adoption, implementation, and technological prospects in the selected countries. A structured critical appraisal was used to assess potential biases in the included studies’ design, selection criteria, and reporting, while findings were thematically analysed to provide actionable and comparative insights. **Results:** The findings reveal that South Africa has the highest adoption rate, focusing on specialist teleconsultations, chronic disease management, and mental health services. Kenya demonstrates strong mHealth integration and advanced mobile applications, particularly in maternal health, HIV care, and sexual and reproductive health. While facing infrastructural and regulatory constraints, Nigeria is advancing innovations for remote diagnosis and teleconsultation. **Conclusions:** By synthesising evidence from peer-reviewed literature, the review identifies adoption trends, enabling factors, and opportunities for scaling telemedicine in these contexts. Despite these advancements, challenges persist, including regulatory gaps, digital literacy limitations, and infrastructure constraints. Addressing these barriers requires targeted investments in broadband expansion, policy harmonisation, and healthcare workforce training to optimise telemedicine’s impact and ensure its sustainability as a healthcare delivery model in Sub-Saharan Africa.

## 1. Introduction

Access to quality healthcare remains a significant challenge in Sub-Saharan Africa, with enormous disparities in healthcare delivery between urban and rural areas [1,2,3]. Telemedicine, which utilises information and communication technologies (ICTs) to provide healthcare services remotely, has emerged as a promising solution to bridge these gaps. It offers improved healthcare access, efficient resource utilisation, and reduced infection risks [3,4,5,6]. By enabling real-time consultations, diagnostics, and remote monitoring, telemedicine can mitigate traditional barriers such as geographical isolation, limited healthcare infrastructure, and critical shortages of medical professionals. However, its adoption in this region comes with some challenges, including infrastructural deficiencies [7], regulatory constraints [8], and socio-cultural barriers [9,10]. Notably, the COVID-19 pandemic is a pivotal reason for adopting and expanding telemedicine worldwide [11,12]. With social distancing measures and restricted mobility, healthcare systems have been compelled to explore digital solutions to maintain continuity of care.

The deployment of telemedicine worldwide through various technologies across different contexts includes mobile applications (apps) [13] designed for smartphones and tablets; SMS or text messaging [14] for delivering health reminders, treatment adherence support, and health tips; video conferencing [15] for face-to-face communication between patients and healthcare providers; secure web-based portals [4] for access to medical records and virtual consultations; and telephonic communication [16] as a reliable option for follow-ups in areas with limited internet access. Additionally, wearable devices [17,18] and remote patient monitoring (RPM) technologies are also being used to enable continuous tracking of health parameters and real-time data sharing with healthcare providers. Other modern methods include chatbots [19] and AI-powered assistants [20,21] for answering health-related inquiries, email communication for asynchronous discussions, and store-and-forward systems that allow patients to send images or reports for later review by specialists. These telemedicine approaches are versatile and can be tailored to address the specific challenges of healthcare access in different regions and communities.

South Africa, Kenya, and Nigeria are leading nations in Sub-Saharan Africa exploring telemedicine solutions [22], each offering unique contexts within the regional landscape. With its increased healthcare infrastructure, South Africa faces notable disparities in access between urban and rural areas [23]. Kenya has leveraged mobile health (mHealth) platforms, driven by widespread mobile phone penetration and innovative solutions such as the M-Pesa payment system [24]. As Africa’s most populous country, Nigeria combines immense potential with significant obstacles, characterised by a burgeoning tech sector juxtaposed against critical gaps in healthcare infrastructure [25,26].

Despite advancements in digital health, telemedicine adoption remains uneven across Sub-Saharan Africa, particularly in rural and suburban areas [27,28]. Several factors contribute to this disparity, including limited internet connectivity, high implementation costs, and a lack of public awareness regarding digital health solutions. Additionally, regulatory frameworks often lag behind technological progress, creating ambiguity around data privacy, reimbursement models, and cross-border telehealth collaborations. These systemic barriers highlight the need for an integrated approach that aligns technological innovation with policy reforms, infrastructure investment, and capacity building to enable sustainable telemedicine expansion.

This review addresses the following research questions: RQ1: What are telemedicine’s key enablers and prospects in South Africa, Kenya, and Nigeria? RQ2: How do country-specific policies and socio-economic conditions impact telemedicine implementation? RQ3: What role do emerging technologies play in scaling telemedicine solutions in these countries? This review aims to provide a comparative analysis of telemedicine adoption across South Africa, Kenya, and Nigeria, identifying key enablers, challenges, and prospects for scaling digital healthcare solutions. By synthesising evidence from diverse healthcare solutions, this review offers actionable insights for policymakers, healthcare providers, and technology stakeholders, contributing to developing sustainable and equitable telemedicine frameworks in Sub-Saharan Africa.

Unlike previous reviews on telemedicine in Sub-Saharan Africa [2,27,29,30,31,32], which often focus on broad regional trends or single-country [31] analyses, this review provides a comparative, multi-country perspective by specifically examining South Africa, Kenya, and Nigeria, three nations at the forefront of digital health innovations in the region. For instance, Dodoo et al. [27] focused on the barriers to the successful implementation of telemedicine in Sub-Saharan Africa, particularly in the context of the COVID-19 pandemic. Additionally, while many prior studies focus on barriers to telemedicine, this review takes a holistic approach by exploring adoption trends, policy frameworks, and emerging prospects, offering evidence-based recommendations for sustainable scaling.

## 2. Methods

This systematic review was conducted following the Preferred Reporting Items for Systematic Reviews and Meta-Analyses (PRISMA) guidelines and registered with the International Prospective Register of Systematic Reviews (PROSPERO - ID: CRD420251003337) to ensure methodological rigour and transparency. The review centred on telemedicine adoption, implementation, and technological prospects in South Africa, Kenya, and Nigeria. These countries were selected due to their significant contributions to healthcare innovation and technological progress within Sub-Saharan Africa. Notably, Nigeria, Kenya, and South Africa are key examples of economic communities (ECOWAS (https://ecowas.int/ (accessed on 15 January 2025)), EAC (https://www.eac.int/ (accessed on 15 January 2025)), and SADC (https://www.sadc.int/ (accessed on 15 January 2025))) within Sub-Saharan Africa where reasonable progress has been made in telemedicine, especially during the COVID-19 pandemic [2].

By employing a systematic search strategy and rigorous inclusion criteria, the review identifies key trends, challenges, prospects and policy directions while providing a robust foundation for future research and advancement in digital health technologies.

### 2.1. Transparency Statement

This systematic review was conducted with a commitment to transparency and methodological rigour. The study adhered to the Preferred Reporting Items for Systematic Reviews and Meta-Analyses (PRISMA) guidelines to ensure clarity and reproducibility. The search strategies, inclusion, and exclusion criteria were predefined and systematically applied to minimise bias.

All decisions related to article selection, quality assessment, and data extraction were conducted independently by two reviewers, with discrepancies resolved through discussion and consensus reached by agreement. The data sources, search terms, and filtering processes have been explicitly documented to facilitate replication. The paper is a complete, concise, and precise study representation.

### 2.2. Search Strategy

A search was conducted using three databases: PubMed, Scopus, and Web of Science between 22 November and 15 December 2024. Search terms included variations of telemedicine, telehealth, mHealth, and digital health to ensure comprehensive coverage of the topic. The geographical focus was on South Africa, Kenya, and Nigeria within Sub-Saharan Africa. Boolean operators were used to combine keywords, i.e., (“telemedicine” OR “telehealth” OR “mHealth” OR “digital health”) AND (“South Africa” OR “Kenya” OR “Nigeria” OR “Sub-Saharan Africa”) AND (“adoption” OR “implementation” OR “policy”).

For PubMed, the search was restricted to full-text articles published in English from 2014 to 2024, with article types including case reports, classical articles, clinical studies, technical reports, and introductory journal articles. Scopus searches were limited to studies published between 2014 and 2024 in Medicine and Computer Science. Web of Science searches covered articles and proceedings papers from 2014 to 2024. Filters were applied to include only articles written in English. A total of 567 articles were retrieved across the databases (PubMed: 157, Scopus: 317, Web of Science: 93).

### 2.3. Exclusion Criteria

To ensure the inclusion of high-quality and relevant studies in the review, specific exclusion criteria (Table 1) were applied during the screening process. First, duplicate records identified across databases were removed to eliminate redundancy (Duplicate Records—DRs). Studies unrelated to telemedicine, digital health technologies, or healthcare delivery in South Africa, Kenya, or Nigeria were excluded (Unrelated Scope—US).

Additionally, editorials, reviews, and study protocols were excluded from the review as they lacked the required results for analysis (Limited Availability—LA). Studies irrelevant to telemedicine’s adoption, implementation, or technological prospects were also excluded (Irrelevant Study Type—IST). Lastly, studies focusing on regions outside Sub-Saharan Africa and those other than South Africa, Kenya, or Nigeria were removed to ensure the geographical specificity of the review (Geographic Mismatch—GM).

After applying these exclusion criteria, 53 articles were retained for further analysis. The eligibility for inclusion in Table 2 ensures that the included studies provide meaningful and reliable insights into telemedicine adoption, implementation and technological prospects in the selected countries.

### 2.4. Quality Assessment

The quality of the included studies was rigorously evaluated based on their alignment with the three primary research questions (RQs) of this review. Each study was appraised to ensure its methodological soundness and ability to address these specific areas of inquiry, using an assessment based on the research questions [46].

For studies relating to RQ1: “What are telemedicine’s key enablers and prospects in South Africa, Kenya, and Nigeria?”, the focus was on assessing the depth of enablers like technological readiness and policy support prospects and also exploring some of the challenges such as infrastructure limitations, cultural challenges, and regulatory issues. For RQ2: “How do country-specific policies and socio-economic conditions impact telemedicine implementation?”, studies were evaluated by examining the interplay between government policies, healthcare infrastructure, and economic conditions. For RQ3: “What role do emerging technologies play in scaling telemedicine solutions in these countries?”, studies were appraised for their exploration of technological advancements and their application in telemedicine (with particular attention to studies addressing the scalability and sustainability of AI and cloud-based solutions).

As described in Table 3, the grouping is based on the primary objective of each study. Still, some studies have secondary objectives addressing other research questions. For example, the study by Cilliers and Stephenson [47] on user acceptance of Telemedicine in Eastern Cape Province, South Africa, potentially addresses RQ2 and RQ1.

The risk of bias in the included studies was assessed based on key methodological factors, including study design, sample size, transparency in reporting, and potential conflicts of interest in each of the studies. Given the diversity of study types, a structured critical appraisal was conducted, considering selection bias, reporting bias, and methodological rigour. They were evaluated for the clarity of inclusion criteria, completeness of reported findings, and potential confounders. While no formal bias assessment tool was applied, the interpretation of findings identified each study’s limitations, and caution was exercised when synthesising results from studies with potential methodological weaknesses. Specifically, any discrepancies in study quality evaluation were resolved through discussion among the reviewers.

### 2.5. Data Extraction

The data extraction process was designed to align with the study’s objectives, guided by the eligibility criteria for inclusion in Table 2. This serves as a foundational framework, ensuring that the selection and analysis of each study remain relevant and methodologically sound. The extracted data focused on several core areas, including study characteristics (authors, year, and geographic focus), intervention details, outcomes/main findings (barriers, enablers, and policy implications), and population demographics (target groups such as healthcare providers, patients, and policymakers). This approach facilitated the systematic collection of comparable and comprehensive data across the studies.

The eligibility criteria inclusion table was pivotal in the study extraction process, as it delineated the parameters for study selection. By adhering to these criteria, the review ensured the inclusion of studies directly relevant to its objectives, excluding those that deviated from the defined scope.

### 2.6. Data Synthesis

The data extracted from the sources were collated and merged to enable analysis from a single data source. The data were synthesised across three key themes: telemedicine innovations and adoption, healthcare delivery and public health impact, and equity, policy, and socioeconomic factors. The themes explore how emerging technologies, such as mHealth platforms and others, transform healthcare delivery in South Africa, Kenya, and Nigeria. Notably, while increasing mobile penetration, international funding, and policy frameworks serve as enablers [16,35,50], challenges such as inadequate infrastructure, digital literacy gaps, and regulatory challenges continue to hinder widespread adoption [22,49]. This review also highlights comparative insights on adoption, challenges and prospects in improving healthcare access, particularly in rural and underserved areas in South Africa, Nigeria and Kenya (Section 4).

The data synthesis aims to aggregate the telemedicine adoption trends, key implementation challenges, and future opportunities to foster actionable insights for policymakers, healthcare providers, and technology stakeholders. The study does not include an analysis of clinical outcomes or cost-effectiveness assessments but focuses on qualitative and thematic insights from the included studies.

## 3. Results

### 3.1. Search Results and Demographic Characteristics

**Figure 1:**
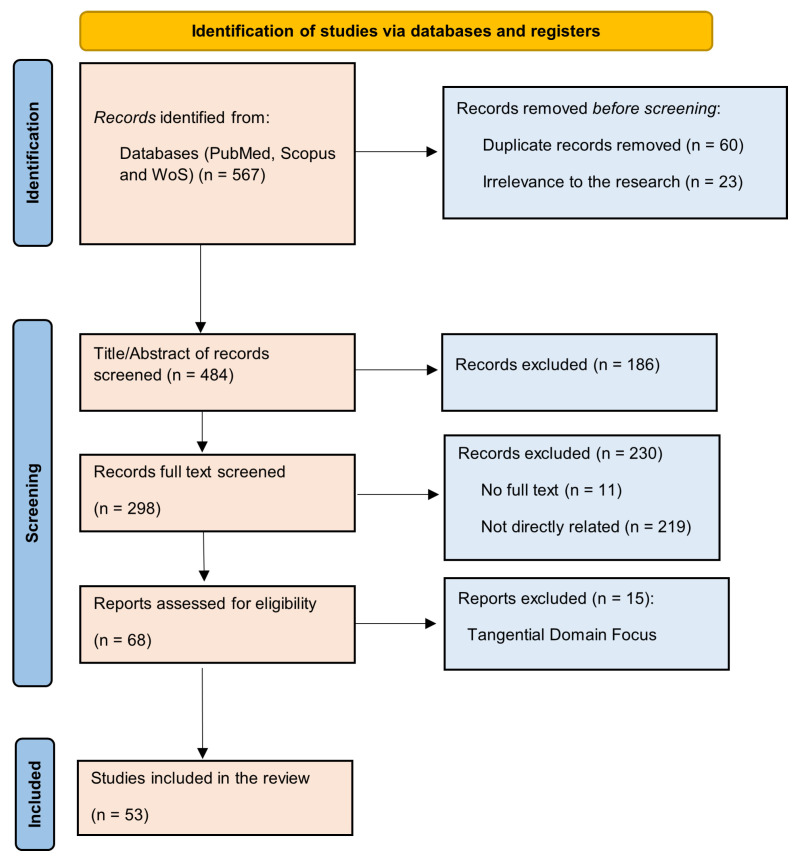
PRISMA statement flow diagram.

The flow diagram (Figure 1) provides a detailed overview of the search and screening process for this systematic review. The search retrieved 567 records from three databases: PubMed, Scopus, and Web of Science (WoS). After removing 60 duplicate entries and 23 records deemed irrelevant to the study’s focus, 484 records proceeded to the title and abstract screening phase. This stage excluded 186 records that did not align with the study objectives, leaving 298 articles for a more in-depth analysis.

During the full-text screening of the 298 articles, 230 records were excluded. This exclusion included 11 articles where full texts were unavailable and 219 articles found to lack direct relevance to the research scope. Following this screening, 68 articles were considered eligible for further assessment. However, an additional 15 reports were excluded at this stage due to their tangential focus or unrelated domains. This comprehensive process (following the exclusion criteria in Table 1) demonstrates a methodical effort to refine the search results and ensure that only the most relevant and high-quality studies were included.

**Figure 2:**
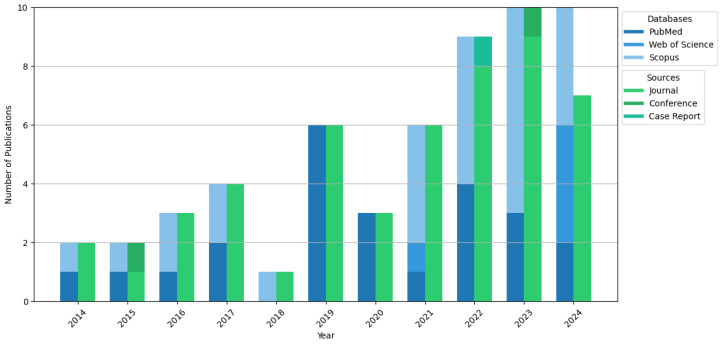
The search source by year of publication, database sources and document types.

In total, 53 studies were selected for inclusion in the review. These included studies had corresponding authors from nine different countries out of the 358 authors and were published within the last 10 years, from 2014 to 2024. Journal articles, case reports, and conference papers are the document types in the included studies. Journal articles comprised the bulk (50 out of 53, or 94.34%). The breakdown is shown in Figure 2.

The included studies reflect diverse geographic and thematic focuses, with significant representation from sub-Saharan Africa. The studies explore the adoption, implementation, prospects and policy frameworks surrounding telemedicine in various healthcare settings. The demographic characteristics and contexts provide critical insights into telemedicine adoption in low-resource environments, offering a robust foundation for discussion and analysis.

### 3.2. Keywords Analysis

**Figure 3:**
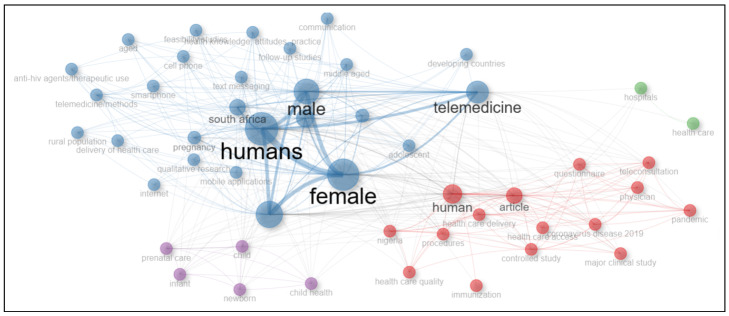
Bibliometric map of included studies (Tool: RStudio, method: keyword Co-occurrence network).

The analysis of keyword co-occurrence was conducted using the RStudio v4.4.2 biblioshiny package [51]. The visualisation of the results, presented in Figure 3, provides critical insights into this study’s thematic areas of interest.

The co-occurrence network diagram identifies keywords central to telemedicine research, such as “telemedicine”, “humans”, “female”, and “male”, which act as highly interconnected hubs. These keywords serve as focal points around various themes and sub-themes. The network is divided into distinct clusters (red, blue, purple and green), each representing areas within the themes.

The blue cluster centres on digital health technologies and their role in enabling healthcare access. Keywords like “telemedicine”, “text messaging”, “mobile applications”, “internet”, “cell phone”, and “delivery of healthcare” are prominent, illustrating the technological enablers critical to scaling telemedicine in Sub-Saharan Africa. The red cluster focuses on healthcare delivery and public health contexts, highlighting keywords such as “human”, “article”, “physician”, “teleconsultation”, “questionnaire”, and “pandemic”. This cluster underscores the importance of telemedicine interventions in addressing healthcare needs for specific populations, particularly women and rural communities. Keywords such as “healthcare access” and “coronavirus disease 2019” suggest a shift toward virtual consultations. The purple cluster highlights the significant role of telemedicine in maternal and neonatal healthcare, emphasising its applications in prenatal care, infant health, and child development. Keywords such as “prenatal care”, “infant”, “child”, “newborn”, and “child health” suggest that telemedicine is being leveraged to enhance access to maternal health services, facilitate remote consultations for expecting mothers, and provide digital tools for early childhood health monitoring. The green cluster underscores the intersection between telemedicine adoption and institutional healthcare frameworks, with the keywords “hospitals” and “health care” focusing on health policy, infrastructure, and institutional readiness.

The connections (edges) between the nodes illustrate the co-occurrence of keywords within the included studies, with thicker edges signifying stronger relationships between key concepts. For instance, “telemedicine” exhibits robust linkages with “health care” and “healthcare delivery”, emphasising its crucial role in enhancing accessibility, efficiency, and continuity of care. Similarly, the term “female” maintains strong associations with “pregnancy” and “child health”, reflecting gender-specific healthcare priorities and the emphasis on maternal health interventions in telemedicine research.

Additionally, cross-thematic interactions between clusters highlight the interplay between digital health solutions, public health strategies, and demographic considerations. The convergence of these areas underscores the multidisciplinary nature of telemedicine research, integrating policy, technology, and health equity frameworks. These interconnections are particularly relevant in low-resource environments across sub-Saharan Africa, where socioeconomic, infrastructural, and healthcare disparities necessitate a holistic and strategic approach to telemedicine adoption and prospects.

### 3.3. Findings

This review synthesised findings from the 53 studies. The findings are presented thematically, highlighting critical enablers, challenges, prospects, and policy implications, particularly addressing health equity challenges and advancing healthcare delivery. The thematic areas revolve around (1) telemedicine innovations and adoption, (2) healthcare delivery and public health impact, and (3) equity, policy, and socioeconomic factors. Then, comparative insights on the adoption, challenges and prospects were highlighted across South Africa, Kenya and Nigeria to understand the varying levels of adoption, policy frameworks and prospects.

#### 3.3.1. Telemedicine Innovations and Adoption

Telemedicine is reshaping healthcare delivery in South Africa, Kenya, and Nigeria, particularly where traditional infrastructure is insufficient. Innovations in mobile health (mHealth) [16,25], mobile applications [52,53], and SMS-based interventions [54,55] are enhancing healthcare accessibility. These digital solutions enable remote consultations, patient education, adherence monitoring, and specialist referrals, improving service delivery across diverse healthcare needs.

The COVID-19 pandemic accelerated telemedicine adoption, prompting the widespread use of instant messaging platforms like WhatsApp for clinical communication and specialist consultations [56]. Additionally, the 2wT (2-way SMS) system facilitated automated patient monitoring and communication in Kenya, although network issues and increased workload for healthcare workers affected its efficiency [54].

Advancements in digital diagnostics and remote patient monitoring have expanded telemedicine’s capabilities. In Nigeria, the Text4Life mHealth platform has improved maternal healthcare accessibility, while patient-held smartcards have streamlined maternal and child health services [25,53]. In South Africa, mobile-based teleconsultation tools have been deployed for burn injury management [57], diabetes care [58], and paediatric epilepsy monitoring [17]. In Kenya, WelTel’s SMS-based intervention has been particularly effective in HIV care engagement [50].

Despite these advancements, telemedicine adoption remains uneven due to infrastructure gaps, regulatory challenges, and digital literacy limitations [47,52,59]. In rural Nigeria, low mobile phone ownership and literacy levels hinder access to mHealth solutions [60]. Kenya’s mobile-first approach has increased adoption, but economic constraints and regulatory uncertainties persist [16,28]. South Africa benefits from a relatively well-developed healthcare infrastructure, but equity gaps remain in underserved regions [47].

The studies also highlight organisational collaborations as crucial for scaling telemedicine. In Kenya, public–private partnerships have enabled AI-driven SMS-based maternal health programs [20]. In Nigeria, the web-based remote patient monitoring system (WB-RPMS) has demonstrated potential in chronic disease management [26]. Innovative models like virtual clinics [59] and mobile health counselling systems [61] are expanding telehealth’s reach.

Telemedicine innovations are bridging healthcare accessibility gaps, improving patient engagement, and enhancing healthcare system efficiency in South Africa, Kenya, and Nigeria (see Table 4). However, the degree of adoption varies by country and is influenced by technological infrastructure, healthcare system maturity, and socio-economic factors.

#### 3.3.2. Healthcare Delivery and Public Health Impact

Telemedicine has significantly transformed healthcare accessibility as emphasised in the studies [6,40,44]. Its integration into healthcare delivery systems has proven effective in enhancing remote patient education, consultation, and treatment. The studies have highlighted mHealth interventions such as WhatsApp-based consultations [19,42,61,62,63,64], SMS-based platforms [14,20,58,65,66,67,68,69,70], and mobile health tools [4,13,15,57,71,72] as essential components in bridging healthcare access gaps. These technologies have been particularly effective during public health crises, including the COVID-19 pandemic, where they alleviated pressure on primary care facilities and facilitated patient monitoring [73].

Mobile-based telemedicine innovations have played a crucial role in addressing specific healthcare challenges. In maternal and child health, Sarna et al. [74] and Owolabi et al. [58] demonstrated how mobile counselling and remote monitoring improved HIV treatment adherence and diabetes management among mothers and infants. Similarly, Adam et al. [71] and Ochieng et al. [20] emphasised that digital interventions enhance maternal knowledge and support postnatal care. Lalla-Edward et al. [13] found that mobile health apps like iThemba Life facilitated real-time access to HIV viral load results, reducing the need for frequent hospital visits. In mental health and psychosocial support, Jarvis et al. [61] demonstrated the effectiveness of WhatsApp-based interventions in reducing loneliness among the elderly. Similarly, Atujuna et al. [65] highlighted mHealth platforms for youth living with HIV. Furthermore, Dulli et al. [75] and Ronen [63] showcased how social media and digital health platforms enhance adherence to ART treatment among youth.

Reproductive health interventions have also benefited from telemedicine. Akande et al. [4] and Johnson et al. [69] discussed the effectiveness of mobile platforms for delivering sexual and reproductive health (SRH) education, reducing barriers related to stigma and geographic inaccessibility. Constant et al. [72] examined self-assessment tools for medical abortion, demonstrating improved autonomy for women seeking reproductive care. Non-communicable disease (NCD) management has also seen a significant impact through telemedicine. Bobrow et al. [14] and Vedanthan et al. [76] showcased how SMS-based interventions enhanced medication adherence for hypertension management. Piotie et al. [77] demonstrated the benefits of nurse-driven, home-based digital interventions for insulin management in type 2 diabetes patients.

The role of AI and chatbots in telemedicine is emerging, with Ochieng et al. [20] exploring AI-enabled SME-based platforms for maternal, newborn and child health (MNCH) in informal settlements. Janssen et al. [5] highlighted an HIV self-testing mobile app, emphasising the importance of privacy and confidentiality in digital health services.

The COVID-19 pandemic further highlighted telemedicine’s role in ensuring continuity of care. Gold-Olufadi et al. [42] emphasised the increased adoption of teledermatology in Nigeria, reducing patient–clinician contact while ensuring effective skin condition management. Blocker et al. [33] highlighted how virtual telemedicine clinics bridged healthcare access in rural and underserved areas.

Despite these advancements, barriers to adoption persist. Nyamu et al. [49] and Amoakoh et al. [78], among others, identified infrastructure limitations, digital illiteracy, and economic constraints as major challenges. Feldacker et al. [67] suggested that strengthening two-way SMS communication could improve medication adherence. Similarly, Lodhia et al. [79] emphasised the need for investment in tele-ophthalmology services to expand specialist care in remote areas.

Telemedicine has played a vital role in transforming healthcare delivery in South Africa, Kenya, and Nigeria, with mHealth platforms and mobile innovations significantly improving healthcare accessibility. However, infrastructural challenges, economic barriers, and digital illiteracy must be addressed to maximise its full potential. Table 5 summarises the insights from the studies.

#### 3.3.3. Equity, Policy, and Socioeconomic Factors of Telemedicine Adoption

For the telemedicine policy frameworks, equity considerations, and socioeconomic factors across South Africa, Kenya, and Nigeria, each country exhibits distinct adoption trends, with Nigeria’s telemedicine growth centred in urban and peri-urban areas through mobile health services [37,44,60], Kenya focusing on rural outreach [28,49,82]. South Africa is leveraging digital health for specialist care, chronic disease management, and mental health support [40,47,83]. However, challenges persist, as shown in Table 6, limiting equitable access and large-scale implementation.

Despite telemedicine’s potential to improve access, it risks exacerbating inequities if digital literacy, affordability, and infrastructure gaps remain unaddressed. Women, elderly individuals, and persons with disabilities often struggle with adoption due to cultural and technological barriers [15,61]. In South Africa, Pillay et al. [83] highlighted that language diversity and digital disparities hinder equitable telemedicine adoption, necessitating policies that improve access for marginalised populations. Similarly, Ikwu et al. [44] emphasised the need for strong legal frameworks to bridge socioeconomic disparities in telemedicine use. In contrast, Gbadamosi et al. [37] underscored affordability as a critical determinant of adoption in low-income communities.

Regulatory gaps further complicate implementation. Endler et al. [40] pointed out that women in low-resource settings face policy-related barriers when accessing telemedicine for reproductive health. Stocks et al. [82] and Salako et al. [84] stressed the need for inclusive policies and digital infrastructure investment to ensure telemedicine reaches disadvantaged groups. Nyamu et al. [49] observed that Kenya recognises the role of its telemedicine strategy in addressing health inequities, yet widespread adoption remains constrained by financial and technical limitations. Similarly, Onsongo et al. [28] noted that policy inconsistencies and limited ICT infrastructure hinder equitable access, particularly in underserved areas.

Institutional readiness plays a key role in adoption. Olufunlayo et al. [60] examined Nigerian tertiary health institutions and found that funding shortages, inadequate infrastructure, and health insurance coverage slow telemedicine adoption. Adenuga et al. [35] identified economic constraints as a significant barrier to clinician participation in telemedicine, while Cilliers and Flowerday [47] noted that rural healthcare workers often lack digital skills, further limiting adoption. Addressing these barriers requires tailored interventions, such as targeted training and incentives for healthcare professionals.

## 4. Comparative Insights on Adoption, Challenges, and Prospects

The adoption, challenges and prospects of telemedicine across South Africa, Nigeria, and Kenya vary significantly as noted from the various thematic insights (Section 3.3.1) due to differences in digital infrastructure, healthcare policies, and socioeconomic factors. While some countries have made substantial progress, others still face barriers to large-scale implementation. However, mobile health (mHealth) interventions, AI-powered telemedicine platforms, and cloud-based health solutions are gaining momentum as viable alternatives to traditional healthcare delivery [20,35,40,49].

### 4.1. South Africa

South Africa exhibits the highest level of telemedicine adoption among the three countries, primarily due to its relatively strong digital health infrastructure and structured healthcare policies [19,47]. The country has successfully integrated telemedicine tools, such as WhatsApp-based platforms, AI-enabled chatbots, and remote patient monitoring systems, into routine healthcare services. Studies highlight the growing use of virtual consultation platforms like Microsoft Teams for specialist consultations and chronic disease management [33].

Several key enablers have contributed to South Africa’s moderate to high adoption levels. These include

- Digital Infrastructure: A strong mobile and broadband network, particularly in urban areas, has facilitated real-time teleconsultations and remote diagnostics [33,57,59].
- Government and Institutional Support: Existing telemedicine policies and professional guidelines, such as those by the Health Professions Council of South Africa (HPCSA), have provided a regulatory framework for telehealth services [83].
- Healthcare Provider Readiness: The integration of mobile health (mHealth) platforms and wearable medical devices into South Africa’s healthcare ecosystem suggests high provider acceptance of telemedicine solutions [13,77].

Notwithstanding these advancements, telemedicine adoption within South Africa remains uneven, with rural and underserved communities experiencing limited access due to infrastructure challenges and affordability concerns [33,40]. Notably, the included studies primarily focus on telemedicine interventions in Gauteng [13,54,65,67,71,77], KwaZulu-Natal [56,61,62,64,83], Western Cape [5,14,17,19,40,57,59,65,70,72], Northern Cape [33], North West [54], and Eastern Cape [47,58]. Among these, Gauteng and Western Cape receive the most attention, likely due to their advanced healthcare infrastructure and greater digital connectivity, which facilitate the adoption of telemedicine solutions. KwaZulu-Natal and Eastern Cape also feature prominently. However, coverage of Northern Cape and North West is minimal, suggesting a need for further exploration of telemedicine in these provinces.

Nonetheless, despite their high rural populations and persistent healthcare access challenges, several provinces, including Limpopo, Mpumalanga, and Free State, are notably not mentioned in the included studies. These regions often experience limited digital infrastructure, lower health workforce availability, and fewer specialist services, making them critical areas for telemedicine expansion [85,86]. Extending digital health initiatives to these underserved areas by strengthening network infrastructure, improving the affordability of telehealth services, and implementing community-based digital literacy programmes is critical to enhancing equitable access to healthcare and ensuring that telemedicine benefits are more evenly distributed across South Africa.

### 4.2. Kenya

Kenya has demonstrated moderate adoption of telemedicine, particularly through mHealth platforms [50,80], AI-enabled SMS interventions [20], and remote consultation [63] services. The country’s strong mobile-first approach to healthcare delivery has enabled cost-effective and scalable digital health interventions, especially for maternal and reproductive health services, infectious disease management, and chronic care [6,66].

Key enablers of telemedicine adoption in Kenya include

- Mobile Health Innovations: Kenya has successfully integrated AI-powered diagnostics, SMS-based maternal health tracking, and mobile-based family planning support into its telemedicine ecosystem [20,63,69].
- Government-Led Initiatives and International Support: Strategic partnerships with global health organisations and government-driven telemedicine programs have accelerated mHealth adoption in rural and underserved regions [64,76].
- Affordable and Scalable Solutions: The preference for low-cost telehealth solutions, such as SMS reminders and voice-based telehealth consultations, has improved accessibility for low-income populations [62,81].

Notably, the included studies on Kenya predominantly focus on urban and peri-urban regions, particularly Nairobi [6,20,28,50,63], Kisumu [16,28,68,74,82], and Mombasa [66], where telemedicine interventions address maternal health, HIV care, and community-based digital health solutions. Other regions such as Eastern Province [49,80], Siaya [68], Nakuru [79], and Uasin Gishu [76,81] are also represented but with fewer studies. However, rural and underserved areas, particularly in Northern Kenya and remote coastal regions, receive little to no coverage in the reviewed literature. This limited focus suggests a gap in telemedicine adoption and implementation in these regions, underscoring the need for expanded research and tailored interventions that address infrastructural limitations and healthcare disparities in resource-constrained settings.

### 4.3. Nigeria

Telemedicine adoption in Nigeria remains low to moderate, primarily due to structural and infrastructural constraints [4,15]. While mHealth interventions have gained traction, the country still lacks a formalised national telemedicine policy, creating uncertainties regarding implementation and provider incentives [60]. However, several digital health initiatives, particularly SMS-based maternal health interventions, mobile health applications, and virtual consultations, are promising to enhance patient engagement and accessibility [35,75].

Key drivers of telemedicine adoption in Nigeria include

- Mobile Technology Penetration: High mobile phone usage has facilitated mHealth adoption, particularly in maternal and child health services [25].
- Community-Based Digital Health Models: Text-based healthcare education platforms and community health worker-driven telemedicine programs have improved outreach in some rural and suburban areas [52,53].
- Private Sector and International Investments: Growing interest in telemedicine from international organisations and technology companies has led to developing technologies such as mhealth applications and telehealth applications for remote consultations [26,52].

The studies highlight telemedicine interventions across various states in Nigeria, including Edo [25], Benue [37,53], Akwa Ibom [75], Enugu [26], Cross River [75], Kwara [4], Lagos [42,84], Oyo [42], FCT [15], and Kebbi [55]. These interventions primarily focus on maternal health, infectious disease management, and digital health solutions. However, despite their sizable populations and healthcare infrastructure challenges, states like Kano and Ogun receive limited attention in the reviewed literature. As a commercial hub with high digital penetration, Lagos presents opportunities for further telemedicine expansion. At the same time, Kano and Enugu, with both urban and rural healthcare disparities, could benefit from targeted digital health interventions, among other states not mentioned (Osun, Kaduna, Anambra, Borno, Sokoto, and the like). The uneven geographic distribution of studies underscores the need for a more inclusive telemedicine research and implementation strategy to ensure equitable healthcare access across Nigeria.

Overall, across South Africa, Kenya, and Nigeria, several common adoption patterns emerge:

- Growing Mobile Health (mHealth) Integration: SMS-based health interventions, smartphone applications, and chatbots are increasing across all three countries.
- Urban–Rural Disparities in Digital Health Access: While urban populations have benefited from broadband-enabled telehealth services, rural communities still struggle with connectivity, affordability, and digital literacy barriers.
- Healthcare Provider Engagement as a Key Adoption Driver: The studies indicate that clinician readiness, training, and institutional support are crucial in determining telemedicine adoption levels.
- Policy and Regulatory Influence on Adoption Rates: Countries with formalised telemedicine policies (such as South Africa) exhibit higher adoption, whereas countries lacking structured frameworks (such as Nigeria and Kenya) face slower telemedicine integration.

Also, some common barriers persist, including the following.

- Limited policy support: The absence of national telemedicine frameworks in Nigeria and Kenya prevents widespread adoption. Even in South Africa, where some guidelines exist, gaps in regulatory structures hinder full-scale implementation.
- Financial constraints: The cost of internet, mobile data, and digital devices remains a significant obstacle, particularly in low-income and rural areas. Many patients and providers cannot afford the necessary technology to engage in telemedicine services.
- Infrastructure limitations: Unreliable electricity, weak broadband connectivity, and a lack of ICT infrastructure hinder telemedicine effectiveness, particularly in remote and underserved communities.
- Healthcare provider resistance: Many clinicians remain reluctant to adopt telemedicine due to workflow disruptions, lack of financial incentives, and concerns over diagnostic accuracy in virtual settings.
- Digital literacy gaps: A lack of patient and provider familiarity with telemedicine tools affects adoption rates, requiring greater digital education and training investment.

Looking forward, several shared opportunities for telemedicine expansion emerge across the countries:

- Policy Reforms and Regulatory Advancements: The development of national telemedicine policies that include clear reimbursement structures, licensing regulations, and data protection laws will drive long-term sustainability and provider adoption. South Africa’s existing framework serves as a model that Nigeria and Kenya can adapt to suit their unique healthcare needs.
- Equity-Focused Interventions: Targeted digital inclusion programs, such as subsidised internet access, community-based telemedicine hubs, and digital literacy training, can improve access to telehealth services in rural and underserved communities.
- Expansion of Broadband and Mobile Infrastructure: Investments in broadband expansion, mobile network reliability, and electricity infrastructure will enhance telemedicine accessibility, particularly in remote regions.
- AI and Machine Learning in Telemedicine: Integrating AI-powered diagnostics, predictive analytics, and personalised health recommendations presents new opportunities for scalable, efficient healthcare delivery. AI-driven chatbots and virtual assistants can enhance patient engagement and triage systems, reducing the burden on overstretched healthcare facilities.
- Public–Private Partnerships: Strengthening collaborations between governments, technology firms, and healthcare institutions can accelerate investment in digital health infrastructure, subsidise access to telemedicine services, and support healthcare workforce training.
- Incentivising Healthcare Providers: Capacity-building programs and financial incentives for healthcare providers will improve digital literacy and encourage telemedicine integration into mainstream healthcare workflows.

**Figure 4:**
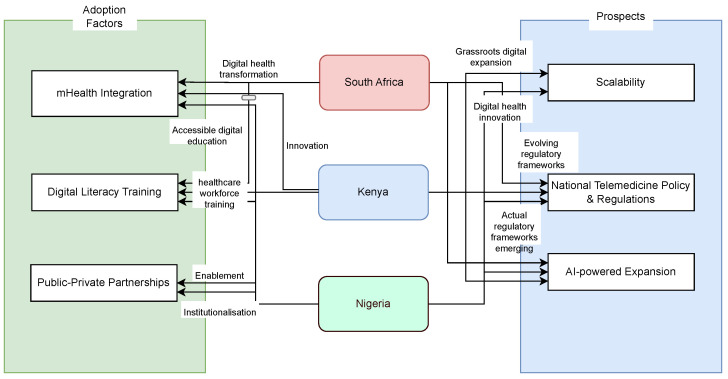
Comparative insights: adoption and prospects across South Africa, Kenya, and Nigeria.

Nonetheless, telemedicine adoption across South Africa, Nigeria, and Kenya is progressing at different rates, influenced by infrastructure readiness, policy frameworks, and healthcare provider engagement. While South Africa leads in adoption, Kenya’s mobile-first approach and Nigeria’s emerging mHealth innovations highlight the transformative potential of digital health solutions. Addressing infrastructure gaps, provider training, affordability constraints, and policy limitations will accelerate widespread telemedicine adoption and ensure equitable access to digital healthcare across sub-Saharan Africa. Table 7 presents an overview of the adoption, policy and implementation. Also, in Figure 4, the illustration of the insights from the countries is presented, showcasing adoption factors and prospects.

Likewise, the acceptance of telemedicine in South Africa, Kenya, and Nigeria is shaped by various socio-cultural factors, including trust in digital health services, gender norms, and traditional healthcare practices. In many rural communities, scepticism toward remote consultations persists due to concerns over misdiagnosis and the impersonality of virtual care [44]. Gender-related barriers also impact access, particularly in conservative societies where women may face restrictions on engaging with male doctors via telehealth platforms [56]. Additionally, the strong reliance on traditional medicine in certain regions may limit the adoption of digital healthcare, as patients often seek guidance from traditional healers before consulting formal healthcare providers [25]. Addressing these challenges through culturally sensitive awareness campaigns, community engagement, and integrating trusted local healthcare figures into telemedicine initiatives could enhance adoption and acceptance.

## 5. Discussion

The findings of this systematic review provide a comprehensive understanding of telemedicine adoption, healthcare delivery impact, and the influence of policy, equity, and socioeconomic factors in South Africa, Kenya, and Nigeria. The results highlight significant progress in telemedicine integration, driven by mobile health (mHealth) technologies, digital platforms, and government-backed initiatives. However, several challenges persist, including infrastructure gaps, digital literacy barriers, and regulatory constraints, which limit telemedicine’s full potential. This discussion synthesises the key insights from the studies, integrating comparative adoption trends, implementation barriers, and prospects.

The adoption varies across South Africa, Nigeria, and Kenya, shaped by the availability of digital infrastructure, healthcare policies, and public–private partnerships. South Africa demonstrates relatively high telemedicine adoption, leveraging mobile applications, WhatsApp-based platforms, and video consultations to enhance healthcare delivery [19,56]. With an internet penetration rate of 74.7% as of January 2024 (https://datareportal.com/reports/digital-2024-south-africa (accessed on 2 February 2025)), South Africa benefits from well-established broadband infrastructure, supporting real-time virtual consultations [83]. Nigeria, in contrast, experiences moderate adoption, largely through SMS-based mHealth interventions such as the IRISS platform and Text4Life [25,52]. Although Nigeria has a high mobile phone penetration rate (over 150 million users), the internet penetration rate stood at 45.5% of the total population at the start of 2024 (https://datareportal.com/reports/digital-2024-nigeria (accessed on 2 February 2025)), low digital literacy and inconsistent broadband access remain barriers [53]. With a growing focus on low-cost telemedicine solutions, Kenya has prioritised SMS-based and AI-enabled interventions, particularly in maternal and child health programs [20,50]. The WelTel platform, for instance, has demonstrated effectiveness in improving patient engagement and adherence [16].

The reviewed studies indicate a positive impact of telemedicine on healthcare delivery, particularly with some statistical results in improving patient adherence, recovery, treatment retention, and accessibility in underserved areas. For instance, HIV care interventions using mobile health (mHealth) solutions in South Africa, Kenya, and Nigeria reported improved retention rates among youth living with HIV, with adherence increasing by approximately 60% [64] retention in care and a 74% [66] viral load suppression rate in some programmes. Similarly, maternal health interventions such as SMS-based intervention systems in Kenya resulted in a 20% increase in mothers seeking care [20]. However, comprehensive longitudinal studies quantifying direct health recovery rates remain limited, highlighting a gap to explore.

Specifically, despite the transformative potential of telemedicine, several structural and policy-related challenges hinder widespread adoption. Infrastructure limitations, particularly unreliable internet connectivity and power supply, are critical barriers in Nigeria and Kenya [28,42]. South Africa, while having a relatively better ICT infrastructure, still faces issues of digital divide and healthcare accessibility in rural communities [40].

On gender and equity disparity, studies highlight that women, elderly individuals, and individuals in low-income regions often experience disparities in telemedicine access due to limited digital literacy and technology affordability [40,61]. Furthermore, the lack of a formalised telemedicine regulatory framework in Nigeria and Kenya restricts large-scale implementation [28,44]. South Africa’s Health Professions Council (HPCSA) provides some guidelines for telemedicine, but there is still a need for clearer reimbursement policies and data protection measures [83].

Data accessibility and protection as emphasised by Pillay et al. [83] remain another critical concern in telemedicine adoption, particularly regarding the protection of electronic health records (EHRs). Several studies [44,60] highlight the risks associated with data breaches, lack of encryption, and inadequate cybersecurity policies. The absence of unified regulatory frameworks across African nations exacerbates these vulnerabilities. Addressing these concerns requires the implementation of robust encryption standards, multi-factor authentication, and region-specific legal frameworks to ensure compliance with global health data protection policies, such as the General Data Protection Regulation (GDPR).

While telemedicine enhances healthcare accessibility, concerns about misdiagnosis can occur due to reliance on remote assessments and limited diagnostic capabilities in some settings. Studies such as Blocker et al. [33] emphasised that the successful implementation of virtual clinics relies heavily on reliable network connectivity, particularly in remote, rural, and underserved areas where such connectivity may be lacking. These challenges can indirectly contribute to potential issues, including misdiagnosis, if communication or data transmission is compromised. Similarly, Ikwu et al. [44] highlighted that misdiagnosis in telemedicine can arise due to limited physical examinations, reliance on technology, and challenges in obtaining comprehensive patient history and informed consent, all of which may compromise diagnostic accuracy. AI-assisted diagnostics and improved training for healthcare workers could mitigate these challenges, ensuring greater reliability in remote healthcare delivery. Conversely, despite its advantages, telemedicine cannot fully replace in-person consultations for severe or complex conditions requiring manual intervention. Hybrid healthcare models that integrate telemedicine with direct clinical interventions are important in such cases requiring in-person intervention.

Likewise, effective feedback mechanisms are essential for improving telemedicine services. In South Africa, feedback from HIV care apps such as iThemba Life received positive patient feedback, with 93% reporting that it was very helpful, 85.5% finding it easy to understand the results, and 97.3% wanting to continue using it for HIV VL results [13]. Studies in Kenya and Nigeria show that mobile health apps implementing two-way feedback loops achieved a response rate of 54.9% [6], with 52.4% [55], allowing healthcare providers to tailor interventions more effectively. However, feedback mechanisms remain underutilised in some areas due to digital literacy barriers and reluctance to engage in post-consultation evaluations.

### 5.1. Recommendations and Future Directions

To optimise telemedicine adoption and the impact of healthcare delivery, the following recommendations are proposed:

- Strengthening Policy Frameworks: Governments in Nigeria, Kenya, and South Africa should establish comprehensive national telemedicine policies that include reimbursement models, licensing regulations, and data privacy laws [28,35].
- Infrastructure Development: Investment in broadband expansion, reliable power supply, and digital infrastructure is essential to support telemedicine scalability in rural and underserved regions [49,60].
- Enhancing Digital Literacy: Training programs targeting healthcare providers and patients can improve telemedicine usability and acceptance, particularly among marginalised populations [47,83].
- Public–Private Partnerships: Encouraging collaboration between governments, technology firms, and healthcare institutions can drive investment in telemedicine infrastructure and service delivery [37,82].
- Equity-Driven Strategies: Targeted interventions such as subsidised mobile health services, AI-enabled health assistants, and localised telemedicine platforms should be prioritised to address healthcare disparities [40,61,84].

Future research should focus on assessing the long-term impact of telemedicine on patient outcomes, particularly in managing chronic diseases and maternal healthcare, as follows:

- Longitudinal Impact Studies: Assess the long-term effectiveness of telemedicine interventions in improving health outcomes, particularly for chronic disease management and maternal healthcare.
- Regulatory and Ethical Considerations: Explore data governance, cybersecurity, and patient consent frameworks to ensure ethical and secure telemedicine practices.
- Cultural and Social Acceptance: Research on the socio-cultural factors influencing telemedicine adoption, including trust in digital health solutions, is essential for tailoring interventions to diverse populations.
- Artificial Intelligence (AI) and Emerging Technologies: Investigating the integration of artificial intelligence, wearable health devices, and blockchain for data security can enhance telemedicine effectiveness.

### 5.2. Study Limitations

This study has some limitations that should be acknowledged. First, variations in study methodologies and telemedicine definitions across the reviewed literature may introduce inconsistencies in analysis. Second, the rapidly evolving nature of telemedicine technologies means that some recent advancements may not have been captured comprehensively, particularly those outside the scope of the included studies. Finally, the study relied on secondary data, and primary fieldwork or interviews with healthcare providers and policymakers could provide deeper insights into adoption, challenges and opportunities.

## 6. Conclusions

This systematic review underscores the transformative role of telemedicine in addressing healthcare challenges across South Africa, Kenya, and Nigeria. Telemedicine has demonstrated its potential to enhance healthcare accessibility, improve efficiency, and bridge gaps in service delivery, particularly in resource-constrained settings. The findings reveal that mHealth solutions, teleconsultation services, and AI-driven digital platforms are actively reshaping healthcare delivery, particularly in maternal health, chronic disease management, and infectious disease care. However, disparities in adoption persist, primarily due to infrastructural limitations, digital literacy gaps, economic constraints, and fragmented policy frameworks.

A multi-faceted approach is essential for integrating telemedicine into mainstream healthcare systems. Strengthening digital infrastructure, particularly in underserved areas, alongside capacity-building programs for healthcare professionals, will enhance adoption. Standardised regulatory frameworks and sustainable reimbursement models are crucial for ensuring data privacy, interoperability, and long-term viability. Economic barriers, such as high mobile data costs, must be addressed to improve accessibility for low-income populations. Collaboration between governments, healthcare institutions, and private stakeholders will be key to scaling telemedicine. By overcoming these challenges, telemedicine can drive equitable healthcare access and contribute to universal health coverage in sub-Saharan Africa.

## Figures and Tables

**Table 1:** Study exclusion criteria.

Code	Exclusion Criteria Description	Number of Studies
DR	Duplicates removed to eliminate redundancy.	60
LA	Excluded editorials, reviews, trial protocols, and similar others.	23
US	Excluded studies that did not focus on telemedicine, digital health technologies, or healthcare delivery in the specified countries.	186
IST	Excluded articles that did not address telemedicine’s adoption, implementation, or technological aspects and those without the full text.	230
GM	Excluded studies focusing on regions outside Sub-Saharan Africa or countries other than South Africa, Kenya, and Nigeria.	15

**Table 2:** Eligibility criteria for inclusion.

Criterion	Inclusion	Exclusion	Justification	Examples
**Population**	Healthcare providers, patients, or policymakers in telemedicine interventions.	Studies not involving the target population.	Focuses on stakeholders directly impacted by telemedicine.	Inclusion: Blocker et al. [33]; Exclusion: Opoku et al. [34].
**Context**	Studies on telemedicine adoption, implementation, or policy in healthcare.	Non-healthcare sectors or unrelated contexts.	Ensures relevance to the review’s scope.	Inclusion: Adenuga et al. [35]; Exclusion: Patel et al. [36].
**Concept**	Telemedicine, telehealth, mHealth, or digital health technologies.	Tangential focus on non-healthcare domains.	Aligns with objectives on telemedicine technologies.	Inclusion: Gbadamosi et al. [37]; Exclusion: Barteit et al. [38].
**Language**	Articles in English.	Non-English publications.	Ensures easy accessibility without translation.	Inclusion: Blocker et al. [33]; Exclusion: Ferre et al. [39].
**Geographic Focus**	Sub-Saharan Africa and within South Africa, Kenya, and Nigeria.	Regions outside the specified geographic focus.	Maintains regional specificity.	Inclusion: Endler et al. [40]; Exclusion: Karajeanes et al. [41].
**Type of Source**	Peer-reviewed articles, technical studies, or full-text publications.	Editorials, letters, reviews, or unavailable full texts.	Emphasises methodologically robust studies.	Inclusion: Gold-Olufadi et al. [42]; Exclusion: Onu and Onyeka [43].
**Publication Timeline**	Published between 2014 and 2024.	Articles published before 2014.	Captures recent advancements in telemedicine.	Inclusion: Ikwu et al. [44]; Exclusion: Hu et al. [45].

**Table 3:** Study quality assessment criteria.

RQ	Description	Total Studies/Sample
RQ1	Studies addressing research question 1	10 (Kwateng et al. [48], Nyamu et al. [49], …)
RQ2	Studies addressing research question 2	11 (Endler et al. [40], Gbadamosi et al. [37], …)
RQ3	Studies addressing research question 3	32 (Blocker et al. [33], Macharia et al. [6], …)

**Table 4:** Summary of studies on Telemedicine innovations and adoption (enablers, target population, technology platform, and prospects across uncluded studies) (n = 10).

Study	Year	Country	Technology Platform	Target Population	Enablers	Adoption Level	Prospect
Day et al. [54]	2023	South Africa	Text message (SMS)	Healthcare providers	Digital infrastructure, healthcare readiness, provider engagement	Moderate	Improved access, cost-effectiveness, scalability
Morris et al. [56]	2024	South Africa	WhatsApp	Healthcare providers	Digital infrastructure, system readiness, provider competency	Moderate	Healthcare accessibility, efficiency, service quality
Blocker et al. [59]	2023	South Africa	Microsoft Teams	Patients	Infrastructure reliability, digital competency, system readiness	Moderate	Healthcare accessibility, infrastructure development, service adaptation
Obi-Jeff et al. [52]	2021	Nigeria	Mobile health application (IRISS)	Caregivers and parents	Digital access, literacy barriers, community engagement	Low	Healthcare outreach, community engagement, immunisation adherence
Udenigwe et al. [25]	2022	Nigeria	Text message (SMS)	Patients and healthcare providers	Community acceptance, user engagement, healthcare access	High	Healthcare access, maternal outcomes, service utilisation
Itanyi et al. [53]	2023	Nigeria	Integrated mobile health platform	Patients	Healthcare support, digital literacy, technology acceptance	Moderate	Healthcare accessibility, maternal care
Meffert et al. [16]	2024	Kenya	mHealth (audio-only mobile phone)	Patients	Digital accessibility, service delivery	Moderate	Healthcare access, cost-effectiveness
Smillie et al. [50]	2014	Kenya	mHealth (WelTel text)	Patients	Digital access, cost-efficiency	High	Patient engagement, care continuity, stigma reduction
Obi-Jeff et al. [55]	2022	Nigeria	mHealth (SMS Reminder System)	Patients	Community engagement, healthcare workforce, infrastructure development	Moderate	Healthcare outreach, immunisation adherence, service access
Onyeabor et al. [26]	2024	Nigeria	Web-based App	Patients	Healthcare access, digital solutions, patient engagement	Low	Healthcare accessibility, cost-effectiveness, system resilience

**Table 5:** Summary of studies on healthcare delivery and public health impact (analysis of telemedicine interventions, public health outcomes, and key challenges) (n = 32).

Study	Year	Country	Focus	Intervention	Adoption Level	Challenges
Mash et al. [19]	2022	South Africa	Diabetes management	mHealth (WhatsApp Chatbot)	Moderate	Digital Literacy, Infrastructure Issues, Technical Challenges, Sustainable Engagement.
Khan et al. [15]	2022	Nigeria	Endocrinology	Teleconsultation (video)	Moderate	Infrastructure Limitations, Digital Literacy, Data Protection and Privacy Concerns.
Harder et al. [80]	2020	Kenya	Mental health (AUDs)	mHealth (phone calls)	Moderate	Infrastructure barriers, literacy gaps, social stigma.
Sarna et al. [74]	2019	Kenya	Maternal health (PMTCT)	mHealth (phone calls)	High	Access barriers, contact challenges, scheduling limitations.
Owolabi et al. [58]	2019	South Africa	Diabetes management	mHealth (text messaging)	High	Digital barriers, literacy gaps, socioeconomic challenges
Hasselberg et al. [57]	2017	South Africa	Acute burns	Teleconsultation (Smartphone app)	Moderate	Infrastructure barriers, digital competency, resource constraints
Jarvis et al. [61]	2019	South Africa	Mental health (LI-CBT)	mHealth (WhatsApp)	Low	Technology barriers, participant retention, digital literacy.
Akande et al. [4]	2024	Nigeria	Sexual and reproductive health (SRH)	Web-based App	High	Privacy concerns, access barriers, socioeconomic factors.
Adam et al. [71]	2023	South Africa	Maternal health	mHealth (SAS videos)	High	Technology access, digital literacy, infrastructure barriers.
Lalla-Edward et al. [13]	2022	South Africa	HIV care	mHealth (mobile App)	High	Access barriers, technical limitations, connectivity issues.
Zunza et al. [70]	2017	South Africa	Maternal health	mHealth (text messaging)	Moderate	Healthcare resources, provider training, participant engagement.
Johnson et al. [69]	2017	Kenya	Family planning and reproductive health	mHealth (text messaging)	Moderate	Demographic bias, evaluation barriers.
Davies et al. [17]	2021	South Africa	Paediatric epilepsy	Wearable devices & App	Moderate	Technology access, security concerns, literacy barriers
Constant et al. [72]	2014	South Africa	Maternal health	mHealth (chat application & USSD)	Moderate	Digital literacy, infrastructure reliability.
Bobrow et al. [14]	2014	South Africa	Hypertension management	mHealth (SMS)	Moderate	Limited engagement, digital literacy.
Macharia et al. [6]	2022	Kenya	Sexual reproductive health (SRH)	mHealth (USSD-based app)	Moderate	Economic barriers, Privacy concerns, Digital literacy.
Janssen et al. [5]	2020	South Africa	HIV care	mHealth (mobile App)	Moderate	Technology access, literacy barriers, security concerns.
Ochieng et al. [20]	2024	Kenya	Maternal, newborn, and child health (MNCH)	AI-enabled SMS-based platform	High	Infrastructure, digital literacy, stakeholder engagement.
Atujuna et al. [65]	2021	South Africa	HIV care	mHealth (SMS)	Moderate	Infrastructure barriers, access limitations, digital literacy
Blocker 2024 [33]	2024	South Africa	Primary healthcare	Virtual clinic system (web-based app)	Moderate	Infrastructure barriers, digital literacy, implementation challenges.
Kurth et al. [81]	2019	Kenya	HIV care	mHealth (mobile App)	High	Infrastructure barriers, stigma concerns, provider resistance.
Zanoni et al. [64]	2024	South Africa	HIV care	mHealth (WhatsApp)	Moderate	Technology barriers, attendance challenges, infrastructure issues.
Piotie et al. [77]	2021	South Africa	Diabetes management	mobile App	Moderate	Infrastructure barriers, provider resistance, resource limitations.
Harrington et al. [68]	2019	Kenya	Maternal health	mHealth (SMS)	High	Partner engagement, recruitment challenges, reporting bias.
Dulli et al. [75]	2020	Nigeria	HIV care	Social media (Facebook)	High	Digital literacy, Supportive infrastructure.
Vedanthan et al. [76]	2019	Kenya	Hypertension care	mHealth (mobile App)	Moderate	Demographic inconsistencies, Economic barriers, Lack of health insurance.
Bergam et al. [62]	2019	South Africa	HIV care	mHealth (WhatsApp)	High	Technology access, digital literacy, connectivity barriers.
Feldacker at al. [67]	2023	South Africa	Voluntary medical male circumcision (VMMC)	mHealth (SMS)	Moderate	Technology barriers, provider literacy, patient confidence.
Lodhia et al. [79]	2016	Kenya	Ophthalmic health	mHealth (mobile App)	Moderate	Infrastructure barriers, digital literacy, sustainability challenges.
Gold-Olufadi et al. [42]	2023	Nigeria	Dermatology care	Teledermatology (WhatsApp)	Moderate	Infrastructure barriers, awareness gaps, cost limitations
Ronen et al. [63]	2023	Kenya	HIV care	mHealth (WhatsApp)	High	Technology access, digital literacy, implementation barriers
Aunon et al. [66]	2023	Kenya	HIV care	mHealth (SMS)	High	Technology access, recruitment barriers, literacy gaps

**Table 6:** Summary of studies on equity, policy, and socioeconomic factors of telemedicine adoption (analysis of policy frameworks, socioeconomic influences, equity considerations, and challenges affecting telemedicine adoption) (n = 11).

Study	Year	Country	Policy Focus	Socioeconomic Factors	Equity Considerations	Challenges	Recommendations
Pillay et al. [83]	2021	South Africa	Health Professions Council of South Africa (HPCSA) telemedicine guidelines	Affordability, digital literacy, infrastructure access	Yes (rural and underserved communities, low-income populations, and ethnic and linguistic minorities)	Infrastructure costs, technology access, digital literacy	Policy reforms.
Ikwu et al. [44]	2021	Nigeria	No	Affordability, digital literacy, inadequate infrastructure	Yes (rural and underserved communities)	No regulation, network issue, electricity issue, cultural beliefs	Legislation and advocacy.
Endler et al. [40]	2022	South Africa	No	Affordability, digital literacy, access to technology	Yes (women, low-income populations, ethnic and linguistic minorities, rural and underserved communities)	Digital access, literacy barriers, systemic constraints	Guided self-assessment protocols.
Gbadamosi et al. [37]	2018	Nigeria	No	Affordability, digital literacy, internet connectivity, health infrastructure	Yes (rural and underserved communities, low-income populations, and women)	Lack of reliable internet connectivity, affordability of technology, digital literacy, inadequate health infrastructure, and policy constraints	Integrated mHealth platforms.
Stocks et al. [82]	2022	Kenya	No	Affordability, digital literacy, and infrastructure	Yes (rural and underserved communities, low-income populations, and ethnic and linguistic minorities)	Infrastructure, financial resources, inadequate training and support	Context-specific development.
Salako et al. [84]	2016	Nigeria	No	Affordability, digital literacy, and infrastructure	Yes (rural and underserved communities)	Socioeconomic status, technology adoption, system integration	Public awareness programs.
Nyamu et al. [49]	2015	Kenya	No	Affordability, digital literacy, ICT infrastructure, and economic levels	Yes (rural and underserved communities)	Infrastructure limitations, cost barriers, resource scarcity	Organisational collaboration.
Onsongo et al. [28]	2023	Kenya	No	Infrastructure costs, digital literacy, resource limitations	Yes (rural and underserved communities, low-income populations, elderly populations, and people living with chronic disease)	Infrastructure limitations, provider readiness, regulatory gaps	Policy reform.
Olufunlayo et al. [60]	2023	Nigeria	No	Affordability, inadequate infrastructure, and digital literacy	Yes (rural and underserved communities, low-income populations, and elderly populations)	Infrastructure gaps, funding limitations, regulatory constraints	Policy reforms and capacity building.
Adenuga et al. [35]	2017	Nigeria	No	Affordability, digital literacy, and infrastructure	Yes (rural and underserved communities, low-income populations, and people living with chronic diseases)	Lack of reimbursement policy, erratic internet connectivity, and inadequate infrastructure	Reimbursement policy.
Cilliers and Flowerday [47]	2014	South Africa	No	Access to technology, digital literacy, affordability	Yes (rural and underserved communities)	Unreliable electricity supply, poor internet connectivity, digital literacy	Education and training.

**Table 7:** Telemedicine adoption, Ccallenges and prospects across South Africa, Kenya, and Nigeria (summary of regulatory frameworks, infrastructure, equity, digital literacy, economic factors, and opportunities).

Aspect	South Africa	Kenya	Nigeria
Policy Frameworks	Existing telemedicine guidelines (HPCSA) require updates for modern integration	No formal national policy; regulatory gaps hinder large-scale adoption	Absence of a national framework; weak enforcement mechanisms
Equity and Access	Digital divide remains a challenge, particularly in rural areas	High costs and limited access to technology restrict widespread adoption	Socioeconomic disparities and infrastructure gaps limit equitable access
Infrastructure	Fair internet coverage, but connectivity issues persist in underserved areas	Unstable power supply and limited broadband availability	Poor network coverage and frequent power outages hinder telemedicine adoption
Private Sector Role	Private investment is increasing, but policy gaps restrict full-scale expansion	Limited private sector engagement; greater public–private partnerships needed	Some government-backed initiatives exist, but long-term sustainability is uncertain
Digital Literacy	Inadequate digital literacy among healthcare providers and users	Insufficient digital skills impede telemedicine adoption	Limited digital literacy remains a key barrier to implementation
Economic Factors	Telemedicine costs vary; affordability remains a barrier for low-income populations	High data and device costs limit adoption, particularly in rural communities	High mobile data costs and limited financial incentives slow adoption
Opportunities	Policy reforms, digital incentives, and improved connectivity could drive expansion	Government support, mHealth integration, and USSD-based solutions offer potential	Strengthened public–private partnerships could enhance access and sustainability

## Data Availability

Not applicable.

## References

[1] Agyepong I.A., Sewankambo N., Binagwaho A., Coll-Seck A.M., Corrah T., Ezeh A., Fekadu A., Kilonzo N., Lamptey P., Masiye F. (2017). The path to longer and healthier lives for all Africans by 2030: The Lancet Commission on the future of health in sub-Saharan Africa. Lancet.

[2] Dodoo J.E., Al-Samarraie H., Alsswey A. (2022). The development of telemedicine programs in Sub-Saharan Africa: Progress and associated challenges. Health Technol..

[3] Mbunge E., Muchemwa B., Batani J. (2022). Are we there yet? Unbundling the potential adoption and integration of telemedicine to improve virtual healthcare services in African health systems. Sens. Int..

[4] Akande O.W., Muzigaba M., Igumbor E.U., Elimian K., Bolarinwa O.A., Musa O.I., Akande T.M. (2024). The effectiveness of an m-Health intervention on the sexual and reproductive health of in-school adolescents: A cluster randomized controlled trial in Nigeria. Reprod. Health.

[5] Janssen R., Engel N., Esmail A., Oelofse S., Krumeich A., Dheda K., Pai N.P. (2020). Alone but supported: A qualitative study of an HIV self-testing app in an observational cohort study in South Africa. AIDS Behav..

[6] Macharia P., Pérez-Navarro A., Sambai B., Inwani I., Kinuthia J., Nduati R., Carrion C. (2022). An Unstructured Supplementary Service Data–Based mHealth App Providing On-Demand Sexual Reproductive Health Information for Adolescents in Kibra, Kenya: Randomized Controlled Trial. JMIR Mhealth Uhealth.

[7] Alegbeleye B.J., Mohammed R.K. (2020). Challenges of healthcare delivery in the context of COVID-19 pandemic in Sub-Saharan Africa. Iberoam. J. Med..

[8] Gaobotse G., Mbunge E., Batani J., Muchemwa B. (2022). The future of smart implants towards personalized and pervasive healthcare in Sub-Saharan Africa: Opportunities, barriers and policy recommendations. Sens. Int..

[9] Ahinkorah B.O., Budu E., Seidu A.A., Agbaglo E., Adu C., Ameyaw E.K., Ampomah I.G., Archer A.G., Kissah-Korsah K., Yaya S. (2021). Barriers to healthcare access and healthcare seeking for childhood illnesses among childbearing women in sub-Saharan Africa: A multilevel modelling of Demographic and Health Surveys. PLoS ONE.

[10] Nyande F.K., Ricks E., Williams M., Jardien-Baboo S. (2022). Socio-cultural barriers to the delivery and utilisation of child healthcare services in rural Ghana: A qualitative study. BMC Health Serv. Res..

[11] Contreras C.M., Metzger G.A., Beane J.D., Dedhia P.H., Ejaz A., Pawlik T.M. (2020). Telemedicine: Patient-Provider Clinical Engagement During the COVID-19 Pandemic and Beyond. J. Gastrointest. Surg..

[12] Nittari G., Savva D., Tomassoni D., Tayebati S.K., Amenta F. (2022). Telemedicine in the COVID-19 Era: A Narrative Review Based on Current Evidence. INternational J. Environ. Res. Public Health.

[13] Lalla-Edward S.T., Mashabane N., Stewart-Isherwood L., Scott L., Fyvie K., Duncan D., Haile B., Chugh K., Zhou Y., Reimers J. (2022). Implementation of an mHealth App to Promote Engagement During HIV Care and Viral Load Suppression in Johannesburg, South Africa (iThemba Life): Pilot Technical Feasibility and Acceptability Study. JMIR Form. Res..

[14] Bobrow K., Farmer A.J., Springer D., Shanyinde M., Yu L.M., Brennan T., Rayner B., Namane M., Steyn K., Tarassenko L. (2016). Mobile phone text messages to support treatment adherence in adults with high blood pressure (StAR): A single-blind, randomized trial. Circulation.

[15] Khan Z., Mlawa G., Yousif Y., Afghan A., Balami D., Mohammed M., Muhammad S., Warrier V., Gupta A., Ibrahim M. (2022). The future of cross-continental telemedicine in the management of complicated endocrine patients and its suitability based on a case report. Cureus.

[16] Meffert S., Mathai M., Neylan T., Mwai D., Onyango D.O., Rota G., Otieno A., Obura R.R., Wangia J., Opiyo E. (2024). Preference of mHealth versus in-person treatment for depression and post-traumatic stress disorder in Kenya: Demographic and clinical characteristics. BMJ Open.

[17] Davies E.H., Fieggen K., Wilmshurst J., Anyanwu O., Burman R.J., Komarzynski S. (2021). Demonstrating the feasibility of digital health to support pediatric patients in South Africa. Epilepsia Open.

[18] Koch M., Matzke I., Huhn S., Sié A., Boudo V., Compaoré G., Maggioni M.A., Bunker A., Bärnighausen T., Dambach P. (2023). Assessing the Effect of Extreme Weather on Population Health Using Consumer-Grade Wearables in Rural Burkina Faso: Observational Panel Study. JMIR Mhealth Uhealth.

[19] Mash R., Schouw D., Fischer A.E. (2022). Evaluating the Implementation of the GREAT4Diabetes WhatsApp Chatbot to Educate People with Type 2 Diabetes During the COVID-19 Pandemic: Convergent Mixed Methods Study. JMIR Diabetes.

[20] Ochieng’ S., Hariharan N., Abuya T., Okondo C., Ndwiga C., Warren C.E., Wickramanayake A., Rajasekharan S. (2024). Exploring the implementation of an SMS-based digital health tool on maternal and infant health in informal settlements. BMC Pregnancy Childbirth.

[21] Perez K., Wisniewski D., Ari A., Lee K., Lieneck C., Ramamonjiarivelo Z. (2025). Investigation into Application of AI and Telemedicine in Rural Communities: A Systematic Literature Review. Healthcare.

[22] Akintunde T.Y., Akintunde O.D., Musa T.H., Sayibu M., Tassang A.E., Reed L.M., Chen S. (2021). Expanding telemedicine to reduce the burden on the healthcare systems and poverty in Africa for a post-coronavirus disease 2019 (COVID-19) pandemic reformation. Glob. Health J..

[23] Clouse K., Phillips T.K., Camlin C., Noholoza S., Mogoba P., Naidoo J., Langford R., Weiss M., Seebregts C.J., Myer L. (2020). CareConekta: Study protocol for a randomized controlled trial of a mobile health intervention to improve engagement in postpartum HIV care in South Africa. Trials.

[24] Agbeyangi A., Suleman H. (2024). Advances and Challenges in Low-Resource-Environment Software Systems: A Survey. Informatics.

[25] Udenigwe O., Okonofua F.E., Ntoimo L.F., Yaya S. (2022). Enablers and barriers to the acceptability of mHealth for maternal healthcare in rural Edo, Nigeria. Dialogues Health.

[26] Onyeabor U.S., Okenwa W.O., Onwuasoigwe O., Lasebikan O.A., Schaaf T., Pinkwart N., Balzer F. (2024). Telemedicine in the age of the pandemics: The prospects of web-based remote patient monitoring systems for orthopaedic ambulatory care management in the developing economies. Digit. Health.

[27] Dodoo J.E., Al-Samarraie H., Alzahrani A.I. (2021). Telemedicine use in Sub-Saharan Africa: Barriers and policy recommendations for COVID-19 and beyond. Int. J. Med. Inform..

[28] Onsongo S., Kamotho C., Rinke de Wit T.F., Lowrie K. (2023). Experiences on the Utility and Barriers of Telemedicine in Healthcare Delivery in Kenya. Int. J. Telemed. Appl..

[29] Chigaro S., Ruredzo I.M., Marembo T. (2023). Integration of telehealth systems into HIV care services in sub-Saharan Africa: A scoping review. Texila Int. J. Public Health.

[30] Chitungo I., Mhango M., Mbunge E., Dzobo M., Musuka G., Dzinamarira T. (2021). Utility of telemedicine in sub-Saharan Africa during the COVID-19 pandemic. A rapid review. Hum. Behav. Emerg. Technol..

[31] Dzando G., Akpeke H., Kumah A., Agada E., Lartey A.A., Nortu J., Nutakor H.S., Donyi A.B., Dordunu R. (2022). Telemedicine in Ghana: Insight into the past and present, a narrative review of literature amidst the Coronavirus pandemic. J. Public Health Afr..

[32] Kipruto H., Muneene D., Droti B., Jepchumba V., Okeibunor C.J., Nabyonga-Orem J., Karamagi H.C. (2022). Use of Digital Health Interventions in Sub-Saharan Africa for Health Systems Strengthening Over the Last 10 Years: A Scoping Review Protocol. Front. Digit. Health.

[33] Blocker A., Datay M.I., Mwangama J., Malila B. (2024). Development of a telemedicine virtual clinic system for remote, rural, and underserved areas using user-centered design methods. Digit. Health.

[34] Opoku D., Busse R., Quentin W. (2019). Achieving Sustainability and Scale-Up of Mobile Health Noncommunicable Disease Interventions in Sub-Saharan Africa: Views of Policy Makers in Ghana. JMIR Mhealth Uhealth.

[35] Adenuga K.I., Iahad N.A., Miskon S. (2017). Towards reinforcing telemedicine adoption amongst clinicians in Nigeria. Int. J. Med. Inform..

[36] Patel A.R., Kessler J., Braithwaite R.S., Nucifora K.A., Thirumurthy H., Zhou Q., Lester R.T., Marra C.A. (2017). Economic evaluation of mobile phone text message interventions to improve adherence to HIV therapy in Kenya. Medicine.

[37] Gbadamosi S.O., Eze C., Olawepo J.O., Iwelunmor J., Sarpong D.F., Ogidi A.G., Patel D., Oko J.O., Onoka C., Ezeanolue E.E. (2018). A Patient-Held Smartcard With a Unique Identifier and an mHealth Platform to Improve the Availability of Prenatal Test Results in Rural Nigeria: Demonstration Study. J. Med. Internet Res..

[38] Barteit S., Jahn A., Banda S.S., Bärnighausen T., Bowa A., Chileshe G., Guzek D., Jorge M.M., Lüders S., Malunga G. (2019). E-Learning for Medical Education in Sub-Saharan Africa and Low-Resource Settings: Viewpoint. J. Med. Internet Res..

[39] Ferre Z., Gerstenblüth M., González C., Noboa C., Triunfo P. (2021). Salud y acceso a cuidados médicos durante la pandemia en Uruguay. Rev. Méd. Del Urug..

[40] Endler M., Petro G., Danielsson K.G., Grossman D., Gomperts R., Weinryb M., Constant D. (2022). A telemedicine model for abortion in South Africa: A randomised, controlled, non-inferiority trial. Lancet.

[41] Karajeanes E., Bila D., Luis M., Tovela M., Anjos C., Ramanlal N., Vaz P., Lapão L.V. (2023). The Infomóvel—An information system for managing HIV/AIDS patients in rural areas of Mozambique. BMC Med. Inform. Decis. Mak..

[42] Gold-Olufadi S., Jesuyajolu D., Cole-Adeife O., Emokpare D., Enigbokan O. (2023). Teledermatology During the COVID-19 Pandemic in a Developing Country: Could This Be the Answer to Improving the Reach of Dermatology Care?. Int. J. Dermatol. Venereol..

[43] Onu J.U., Onyeka T.C. (2024). Digital psychiatry in Nigeria: A scoping review. S. Afr. J. Psychiatry.

[44] Ikwu A.N., Komolafe D.T., Ahaneku G.I., Nwawudu S.E. (2021). Advancement of telemedicine in Africa and the current laws: A case study of Nigeria. Med.-Leg. J..

[45] Paul Jen-Hwa Hu P.Y.K.C., Sheng O.R.L. (2002). Adoption of Telemedicine Technology by Health Care Organizations: An Exploratory Study. J. Organ. Comput. Electron. Commer..

[46] Yang L., Zhang H., Shen H., Huang X., Zhou X., Rong G., Shao D. (2021). Quality Assessment in Systematic Literature Reviews: A Software Engineering Perspective. Inf. Softw. Technol..

[47] Cilliers L., Flowerday S. (2014). User Acceptance of Telemedicine by Health Care Workers A Case of the Eastern Cape Province, South Africa. Electron. J. Inf. Syst. Dev. Ctries..

[48] Kwame Owusu Kwateng O.D.L., Amanor K. (2023). A modified UTAUT2 for the study of telemedicine adoption. Int. J. Healthc. Manag..

[49] Nyamu J., De Coster R., Taib S.M. An empirical study of collaborative innovation as a facilitator to telemedicine adoption in developing countries. Proceedings of the 2015 International Conference on Information Society (i-Society).

[50] Smillie K., Borek N.V., Kop M.L.V.D., Lukhwaro A., Li N., Karanja S., Patel A.R., Ojakaa D., Lester R.T. (2014). Mobile health for early retention in HIV care: A qualitative study in Kenya (WelTel Retain). Afr. J. AIDS Res..

[51] Aria M., Cuccurullo C. (2017). bibliometrix: An R-tool for comprehensive science mapping analysis. J. Inf..

[52] Obi-Jeff C., Garcia C., Onuoha O., Adewumi F., David W., Bamiduro T., Aliyu A.B., Labrique A., Wonodi C. (2021). Designing an SMS reminder intervention to improve vaccination uptake in Northern Nigeria: A qualitative study. Bmc Health Serv. Res..

[53] Itanyi I.U., Iwelunmor J., Olawepo J.O., Gbadamosi S., Ezeonu A., Okoli A., Ogidi A.G., Conserve D., Powell B., Onoka C.A. (2023). Acceptability and user experiences of a patient-held smart card for antenatal services in Nigeria: A qualitative study. BMC Pregnancy Childbirth.

[54] Day S., Ncube V., Maja L., Wasunna B., Pienaar J., Setswe G., Waweru E., Feldacker C. (2023). Centering Frontline Health Care Workers in Digital Innovation Design to Inform the Optimization of an App for Improved Male Circumcision Follow-up in South Africa: Qualitative Usability Study. JMIR Form. Res..

[55] Obi-Jeff C., Garcia C., Adewumi F., Bamiduro T., David W., Labrique A., Wonodi C. (2022). Implementing SMS reminders for routine immunization in Northern Nigeria: A qualitative evaluation using the RE-AIM framework. BMC Public Health.

[56] Morris C., Scott R.E., Mars M. (2024). Towards ‘Formalising’ WhatsApp Teledermatology Practice in KZ-N District Hospitals: Key Informant Interviews. Int. J. Environ. Res. Public Health.

[57] Hasselberg M., Lee Wallis P.B., Laflamme L. (2017). A smartphone-based consultation system for acute burns—Methodological challenges related to follow-up of the system. Glob. Health Action.

[58] Owolabi E.O., Goon D.T., Ajayi A.I. (2019). Efficacy, acceptability and feasibility of daily text-messaging in promoting glycaemic control and other clinical outcomes in a low-resource setting of South Africa: A randomised controlled trial. PLoS ONE.

[59] Blocker A., Oladokun A., Datay M.I., Mwangama J., Malila B. Evaluating the Capability of 3G, 4G, and 5G Networks in Delivering a Virtual Clinic Solution. Proceedings of the 2023 IEEE AFRICON.

[60] Olufunlayo T.F., Ojo O.O., Ozoh O.B., Agabi O.P., Opara C.R., Taiwo F.T., Fasanmade O.A., Okubadejo N.U. (2023). Telemedicine ready or not? A cross-sectional assessment of telemedicine maturity of federally funded tertiary health institutions in Nigeria. Digit. Health.

[61] Jarvis M.A., Padmanabhanunni A., Chipps J. (2019). An Evaluation of a Low-Intensity Cognitive Behavioral Therapy mHealth-Supported Intervention to Reduce Loneliness in Older People. Int. J. Environ. Res. Public Health.

[62] Bergam S., Sibaya T., Ndlela N., Kuzwayo M., Fomo M., Goldstein M.H., Marconi V.C., Haberer J.E., Archary M., Zanoni B.C. (2022). “I am not shy anymore”: A qualitative study of the role of an interactive mHealth intervention on sexual health knowledge, attitudes, and behaviors of South African adolescents with perinatal HIV. Reprod. Health.

[63] Ronen K., Mugo C., Kaggiah A., Seeh D., Kumar M., Guthrie B.L., Moreno M.A., John-Stewart G., Inwani I. (2023). Facilitated WhatsApp Support Groups for Youth Living With HIV in Nairobi, Kenya: Single-Arm Pilot Intervention Study. JMIR Form. Res..

[64] Zanoni B.C., Archary M., Sibaya T., Musinguzi N., Gethers C.T., Goldstein M., Bergam S., Psaros C., Marconi V.C., Haberer J.E. (2024). Acceptability, feasibility and preliminary effectiveness of the mHealth intervention, InTSHA, on retention in care and viral suppression among adolescents with HIV in South Africa: A pilot randomized clinical trial. AIDS Care.

[65] Atujuna M., Simpson N., Ngobeni M., Monese T., Giovenco D., Pike C., Figerova Z., Visser M., Biriotti M., Kydd A. (2021). Khuluma: Using Participatory, Peer-Led and Digital Methods to Deliver Psychosocial Support to Young People Living with HIV in South Africa. Front. Reprod. Health.

[66] Aunon F.M., Wanje G., Richardson B.A., Masese L., Odeny T.A., Kinuthia J., Mandaliya K., Jaoko W., Simoni J.M., McClelland R.S. (2023). Randomized controlled trial of a theory-informed mHealth intervention to support ART adherence and viral suppression among women with HIV in Mombasa, Kenya: Preliminary efficacy and participant-level feasibility and acceptability. BMC Public Health.

[67] Feldacker C., Pienaar J., Wasunna B., Ndebele F., Khumalo C., Day S., Tweya H., Oni F., Sardini M., Adhikary B. (2023). Expanding the Evidence on the Safety and Efficiency of 2-Way Text Messaging–Based Telehealth for Voluntary Medical Male Circumcision Follow-up Compared with In-Person Reviews: Randomized Controlled Trial in Rural and Urban South Africa. J. Med. Internet Res..

[68] Harrington E.K., Drake A.L., Matemo D., Ronen K., Osoti A.O., John-Stewart G., Kinuthia J., Unger J.A. (2019). An mHealth SMS intervention on Postpartum Contraceptive Use Among Women and Couples in Kenya: A Randomized Controlled Trial. Am. J. Public Health.

[69] Johnson D., Juras R., Riley P., Chatterji M., Sloane P., Choi S.K., Johns B. (2017). A randomized controlled trial of the impact of a family planning mHealth service on knowledge and use of contraception. Contraception.

[70] Zunza M., Cotton M.F., Mbuagbaw L., Lester R., Thabane L. (2017). Interactive weekly mobile phone text messaging plus motivational interviewing in promotion of breastfeeding among women living with HIV in South Africa: Study protocol for a randomized controlled trial. Trials.

[71] Adam M., Kwinda Z., Dronavalli M., Leonard E., Nguyen V.K., Tshivhase V., Bärnighausen T., Pillay Y. (2023). Effect of Short, Animated Video Storytelling on Maternal Knowledge and Satisfaction in the Perinatal Period in South Africa: Randomized Controlled Trial. J. Med. Internet Res..

[72] Constant D., Katherine de Tolly J.H., Myer L. (2014). Assessment of completion of early medical abortion using a text questionnaire on mobile phones compared to a self-administered paper questionnaire among women attending four clinics, Cape Town, South Africa. Reprod. Health Matters.

[73] Iliyasu Z., Garba R.M., Bashir H.A., Saleh N.S., Jibo A.M., Amole T.G., Umar A.A., Tsiga-Ahmed F.I., Abdullahi H.M., Kwaku A.A. (2024). Telemedicine Service Adoption During the COVID-19 Pandemic: Physicians’ Experience from Nigeria. Telemed. e-Health.

[74] Sarna A., Saraswati L.R., Okal J., Matheka J., Owuor D., Singh R.J., Reynolds N., Kalibala S. (2019). Cell Phone Counseling Improves Retention of Mothers with HIV Infection in Care and Infant HIV Testing in Kisumu, Kenya: A Randomized Controlled Study. Glob. Health Sci. Pract..

[75] Dulli L., Ridgeway K., Packer C., Murray K.R., Mumuni T., Plourde K.F., Chen M., Olumide A., Ojengbede O., McCarraher D.R. (2020). A Social Media–Based Support Group for Youth Living with HIV in Nigeria (SMART Connections): Randomized Controlled Trial. J. Med. Internet Res..

[76] Vedanthan R., Kamano J.H., DeLong A.K., Naanyu V., Binanay C.A., Bloomfield G.S., Chrysanthopoulou S.A., Finkelstein E.A., Hogan J.W., Horowitz C.R. (2019). Community Health Workers Improve Linkage to Hypertension Care in Western Kenya. J. Am. Coll. Cardiol..

[77] Piotie P.N., Wood P., Webb E.M., Hugo J.F., Rheeder P. (2021). Designing an integrated, nurse-driven and home-based digital intervention to improve insulin management in under-resourced settings. Ther. Adv. Endocrinol. Metab..

[78] Amoakoh H.B., Klipstein-Grobusch K., Agyepong I.A., Amoakoh-Coleman M., Kayode G.A., Reitsma J.B., Grobbee D.E., Ansah E.K. (2020). Can an mhealth clinical decision-making support system improve adherence to neonatal healthcare protocols in a low-resource setting?. BMC Pediatr..

[79] Lodhia V., Karanja S., Lees S., Bastawrous A. (2016). Acceptability, Usability, and Views on Deployment of Peek, a Mobile Phone mHealth Intervention for Eye Care in Kenya: Qualitative Study. JMIR mHealth uHealth.

[80] Harder V.S., Musau A.M., Musyimi C.W., Ndetei D.M., Mutiso V.N. (2020). A randomized clinical trial of mobile phone motivational interviewing for alcohol use problems in Kenya. Addiction.

[81] Kurth A.E., Sidle J.E., Chhun N., Lizcano J.A., Macharia S.M., Garcia M.M., Mwangi A., Keter A., Siika A.M. (2019). Computer-Based Counseling Program (CARE+ Kenya) to Promote Prevention and HIV Health for People Living with HIV/AIDS: A Randomized Controlled Trial. AIDS Educ. Prev..

[82] Stocks J., Choi Y., Ibrahim S., Huchko M. (2022). Iterative Development of a Mobile Phone App to Support Community Health Volunteers During Cervical Cancer Screening in Western Kenya: Qualitative Study. JMIR Form. Res..

[83] Pillay L., Govender R., Pillay S. (2021). Doctor-perceived-barriers to telephone clinics at KwaZulu-Natal hospitals during the COVID-19 pandemic. S. Afr. Fam. Pract..

[84] Salako O., Robert A.A., Okunade K.S., Olatunji A., Fakolade A., Isibor V., Falode D. (2016). Utilization of cancer information system for breast cancer control in Lagos, Nigeria. Pan Afr. Med. J..

[85] Balikuddembe J.K., Reinhardt J.D. (2019). Can Digitization of Health Care Help Low-Resourced Countries Provide Better Community-Based Rehabilitation Services?. Phys. Ther..

[86] Sumbana V., Dandadzi T.A., Nkobeni L.M., Ndobe T.V., Seeletse S.M. (2024). The potential value of e-health in a rural Limpopo Province municipality. Int. J. Res. Bus. Soc. Sci..

